# Using Individualized Brain Network for Analyzing Structural Covariance of the Cerebral Cortex in Alzheimer's Patients

**DOI:** 10.3389/fnins.2016.00394

**Published:** 2016-09-01

**Authors:** Hee-Jong Kim, Jeong-Hyeon Shin, Cheol E. Han, Hee Jin Kim, Duk L. Na, Sang Won Seo, Joon-Kyung Seong, Alzheimer's Disease Neuroimaging Initiative

**Affiliations:** ^1^School of Biomedical Engineering, Korea UniversitySeoul, South Korea; ^2^Department of Bio-convergence Engineering, Korea UniversitySeoul, South Korea; ^3^Department of Neurology, Samsung Medical Center, Sungkyunkwan University School of MedicineSeoul, South Korea; ^4^Neuroscience Center, Samsung Medical CenterSeoul, South Korea

**Keywords:** structural covariance network (SCN), individual SCN, Alzheimer's disease, network entropy, network architecture, cortical thickness

## Abstract

Cortical thinning patterns in Alzheimer's disease (AD) have been widely reported through conventional regional analysis. In addition, the coordinated variance of cortical thickness in different brain regions has been investigated both at the individual and group network levels. In this study, we aim to investigate network architectural characteristics of a structural covariance network (SCN) in AD, and further to show that the structural covariance connectivity becomes disorganized across the brain regions in AD, while the normal control (NC) subjects maintain more clustered and consistent coordination in cortical atrophy variations. We generated SCNs directly from T1-weighted MR images of individual patients using surface-based cortical thickness data, with structural connectivity defined as similarity in cortical thickness within different brain regions. Individual SCNs were constructed using morphometric data from the Samsung Medical Center (SMC) dataset. The structural covariance connectivity showed higher clustering than randomly generated networks, as well as similar minimum path lengths, indicating that the SCNs are “small world.” There were significant difference between NC and AD group in characteristic path lengths (*z* = −2.97, *p* < 0.01) and small-worldness values (*z* = 4.05, *p* < 0.01). Clustering coefficients in AD was smaller than that of NC but there was no significant difference (*z* = 1.81, not significant). We further observed that the AD patients had significantly disrupted structural connectivity. We also show that the coordinated variance of cortical thickness is distributed more randomly from one region to other regions in AD patients when compared to NC subjects. Our proposed SCN may provide surface-based measures for understanding interaction between two brain regions with co-atrophy of the cerebral cortex due to normal aging or AD. We applied our method to the AD Neuroimaging Initiative (ADNI) data to show consistency in results with the SMC dataset.

## Introduction

The morphology of cortical gray matter has been widely used for analyzing normal development and aging (Salat et al., 2004; Sowell et al., 2004), degenerative brain diseases (Lerch et al., 2008; Tae et al., 2008; Bernhardt et al., 2009b; Querbes et al., 2009; Koolschijn et al., 2010; Järnum et al., 2011), genetic influence (Panizzon et al., 2009; Winkler et al., 2010), and developmental brain diseases (Shaw et al., 2006, 2007; Hyde et al., 2010; Jiao et al., 2010). Of the various morphological parameters, cortical thickness and gray matter volumetric intensity are representative measures of cortical morphology for gray matter MRI scans. Beyond conventional regional analysis, recent studies have further shown that intracortical similarities in gray matter morphology can provide evidence for structural brain connectivity through the examination of coordinated variations in cortical thickness and volumetric intensity across the brain (Mechelli et al., 2005; Lerch et al., 2006; Seeley et al., 2009; Raznahan et al., 2011; Evans, 2013). These structural covariance networks (SCNs) have been shown to predict anatomical connectivity, similar to diffusion-weighted brain network analysis, both in humans (Lerch et al., 2006), and animals (Barbas, 1986; Barbas and Rempel-Clower, 1997; Dombrowski et al., 2001). It has also been demonstrated that SCNs are sensitive imaging markers for aging (Chen et al., 2008, 2011; Wu et al., 2012), multiple sclerosis (He et al., 2009b), Alzheimer's disease (He et al., 2008, 2009a; Raj et al., 2012; Zhou et al., 2012), schizophrenia (Alexander-Bloch et al., 2012; Zhang et al., 2012), adult/pediatric cancers (Hosseini et al., 2012a,b), and epilepsy (Bernhardt et al., 2008, 2009a, 2011).

Although the previous SCN-based studies represent significant breakthroughs, they are largely reliant on group-level anatomical correlations of cortical morphology (He et al., 2007, 2008; Bassett et al., 2008; Bernhardt et al., 2011; Zalesky et al., 2012; Zhang et al., 2012). Such group-level SCNs have provided a statistical framework to study synchronized morphology changes in brain regions across populations, however, it remains unclear how an individual-level SCN directly from a prospective subject's T1-weighted MR images might be constructed. The morphology of cortical gray matter varies dramatically between individuals (Kennedy et al., 1998; Evans, 2013), therefore, construction of SCNs at the individual level would presumably allow for the direct analysis of individual anatomical structural covariance. In addition, it would facilitate statistical analyses of the theoretical properties of individual SCNs, which is difficult to achieve using group-level SCNs. Previous studies have attempted to construct single-subject SCNs. One such approach proposed a cube-based correlation approach to extract single-subject anatomical connectivity using volumetric intensities (Tijms et al., 2012; Batalle et al., 2013). Rather than the volumetric morphology characteristics, cortical thickness-based individual SCNs have been recently proposed to leverage surface-based sensitive features (Apostolova et al., 2006; Fan et al., 2008; Qiu et al., 2009). For example, Saggar et al. proposed a novel method to estimate individual contributions to group-level SCNs (Saggar et al., 2015), although it cannot be used to construct an individual SCN directly from the T1-weighted MR image. Similarly, some individual anatomical connectivity studies demonstrated an improvement in classification between different groups (Raj et al., 2010; Zhou et al., 2012; Dai et al., 2013; Wee et al., 2013; Tong et al., 2014; Yun et al., 2015; Zheng et al., 2015). These approaches are also limited in part that they require reference models which are usually from a group of normal control subjects to define relative connectivity for an individual subject, or in part that the SCNs were used as features for classification without architecture analysis (Dai et al., 2013; Wee et al., 2013; Tong et al., 2014; Zheng et al., 2015). Recently, Raamana et al. proposed a new methods to construct the individual SCNs using difference of mean cortical thickness between two regions, which is hard to reflect variance of cortical thickness within a region (Raamana et al., 2014, 2015).

In the present study, we sought to extend these studies to construct individual SCNs based on cortical thickness covariance, and applied the proposed approaches to morphometric data from a large group of patients with Alzheimer's disease (AD) and an age and gender-matched group of normal control subjects recruited at Samsung Medical Center (SMC) and applied our method to the Alzheimer's disease Neuroimaging Initiative (ADNI) data to show consistency in results with the SMC dataset. We further hypothesized that the disrupted network architectural properties are caused by “spreading out” of the structural connectivity and examined the hypothesis by investigating randomness of the anatomical covariance connectivity in both groups.

## Materials and methods

### Participants

In this study, we used the SMC dataset. For the SMC dataset, we recruited 379 AD patients between June 2006 and December 2012, and 2231 normal controls (NC) between September 2008 and December 2012 at SMC (Seoul, Republic of Korea). Considering the possibility of amnestic mild cognitive impairment (aMCI) patients included in the same period, we selected 353 AD subjects and 307 NC subjects so that age, education and gender information were matched. We excluded subjects without Mini Mental State Examination (MMSE) scores and images with errors arising from image processing were also excluded. Our final subject pool for the SMC dataset consisted of 205 AD patients and 250 NC subjects. Patients with AD met the diagnostic criteria of the Diagnostic and Statistical manual of the Mental Disorders-Fourth Edition (DSM-IV) (American Psychiatric Association, 1994), and severity was evaluated using the MMSE score. This study was approved by the Institutional Review Board of SMC. We applied our methods to the ADNI dataset to show consistency in results with the SMC dataset. For the ADNI dataset, we used 183 AD patients and 158 age, education and gender matched NC subjects. Data used in the preparation of this article were obtained from the ADNI database (adni.loni.usc.edu). The ADNI was launched in 2003 as a public-private partnership, led by Principal Investigator Michael W. Weiner, MD. The primary goal of ADNI has been to test whether serial magnetic resonance imaging (MRI), positron emission tomography (PET), other biological markers, and clinical and neuropsychological assessment can be combined to measure the progression of mild cognitive impairment (MCI) and early AD.

The demographic and clinical characteristics of the participants are shown in Table 1. In our datasets, age, gender, and education were controlled between two groups. Specifically, age (*t*-test, SMC dataset: *t* = −0.26, *p* = 0.80/ADNI dataset: *t* = −1.53, *p* = 0.13), gender (Chi-square test, SMC dataset: χ^2^ = 3.06, *p* = 0.22/ADNI dataset: χ^2^ = 0.39, *p* = 0.53) and years of education (*t*-test, SMC dataset: *t* = 1.76, *p* = 0.08/ADNI dataset: *t* = 1.78, *p* = 0.08) between the two groups were not statistically different. The MMSE scores (*t*-test, SMC dataset: *t* = 18.9, *p* < 0.01^*^ /ADNI dataset: *t* = 26.1, *p* < 0.01^*^) were significantly different between the two groups.

**Table 1:** **Subject demographic and clinical characteristics**.

		**NC**	**AD**	**Comparison**
SMC	Number of subjects	250	205	
	Age	69.6 ± 8.4	69.8 ± 9.4	*t* = −0.26, *p* = 0.80
	Gender(F/M)	139/111	124/81	χ^2^ = 3.06, *p* = 0.22
	Education	11.5 ± 5.6	10.7 ± 4.9	*t* = 1.76, *p* = 0.08
	MMSE	27.3 ± 2.7	19.2 ± 6.1	*t* = 18.9, *p* < 0.01^*^
ADNI	Number of subjects	158	183	
	Age	76.2 ± 5.4	75.1 ± 7.3	*t* = −1.53, *p* = 0.13
	Gender(F/M)	84/74	90/93	χ^2^ = 0.39, *p* = 0.53
	Education	15.9 ± 2.9	15.4 ± 2.8	*t* = 1.78, *p* = 0.08
	MMSE	29.2 ± 1.0	23.6 ± 2.5	*t* = 26.1, *p* < 0.01^*^

*Normal control (NC) and Alzheimer's disease (AD) subjects were recruited at Samsung Medical Center (SMC). The AD Neuroimaging Initiative (ADNI) dataset was used to show consistency in results with the SMC dataset*.

* *Statistically significant*.

### Image acquisition and preprocessing

In the SMC dataset, three-dimensional T1-weighted Turbo Field Echo magnetic resonance (MR) images were acquired from all 455 subjects (205 AD patients and 250 NC subjects) at SMC using a 3.0T Philips Achieva MRI scanner with the following image parameters: 1 mm sagittal slice thickness, over-contiguous slices with 50% overlap; no gap; repetition time (TR) of 9.9 ms; echo time (TE) of 4.6 ms; flip angle of 8°; and matrix size 240 × 240 pixels, reconstructed to 480 × 480 over a 240 mm field of view.

In the ADNI dataset, T1-weighted MR images were obtained according to a standardized 1.5 Tesla MRI protocol of the ADNI-1 study (Jack et al., 2008). The MR images were acquired with following image parameters: sagittal plane, repetition time/echo time/inversion time 2400/3/1000 ms, flip angle 8°, 24 cm field-of-view, 192 × 192 in-plane matrix, and 1.2-mm slice thickness.

**Figure 1:**
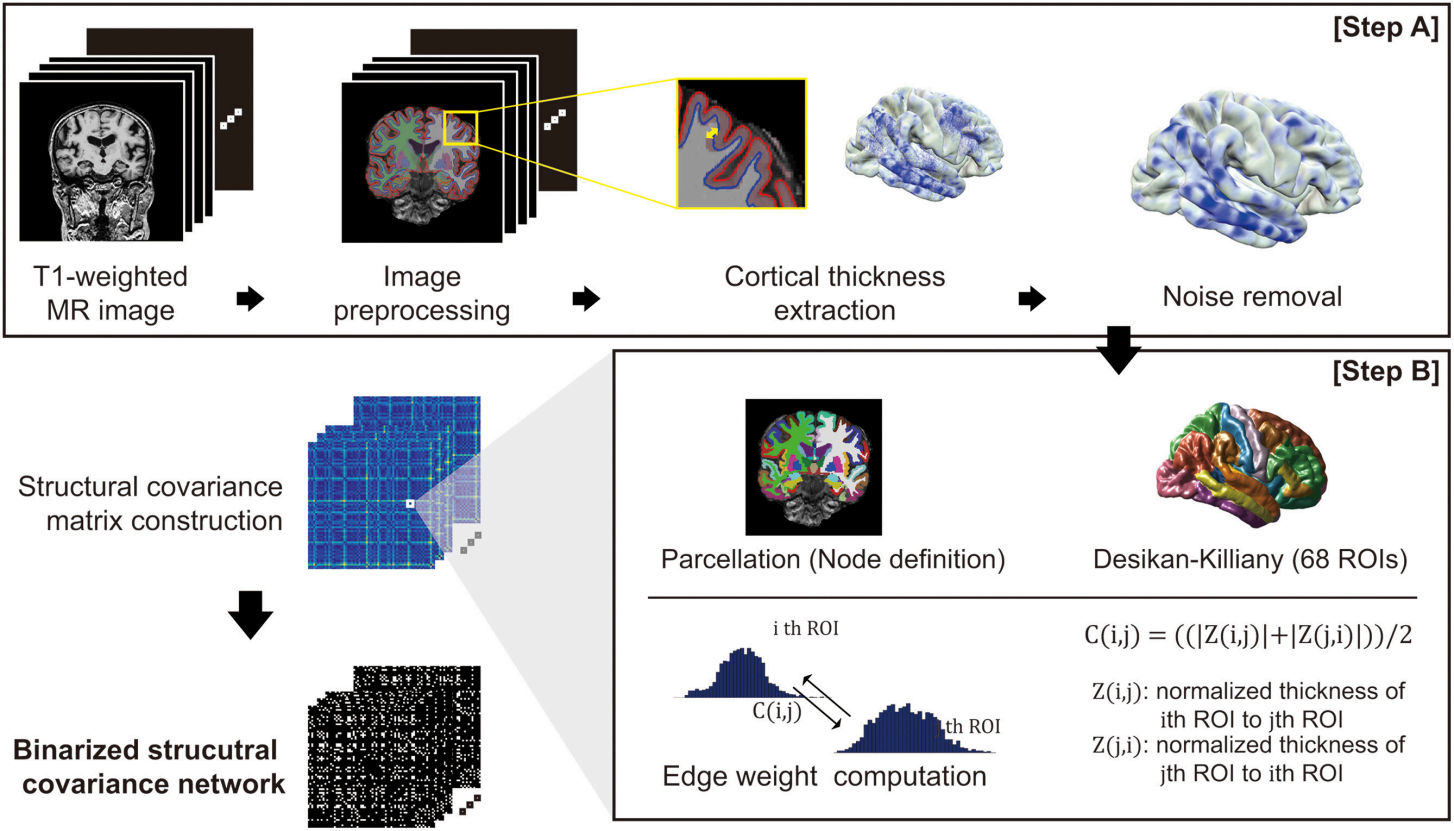
**An overview of the proposed network construction method: T1-weighted MR images undergo an image preprocessing procedure and computation of cortical thickness** (Step A). For the cortical thickness data from each image, the manifold harmonic transform (MHT) is applied to remove noise (Step A). After brain parcellation, the edge weight of the network was computed (Step B), and finally the network is binarized using a threshold.

For each subject, we performed image preprocessing and computed cortical thickness using FreeSurfer v 5.1.0 (http://surfer.nmr.mgh.harvard.edu/) (Step A in Figure 1). Outer and inner cortical surface meshes were first constructed from T1-weighted MR data. The inner surface represented the boundary between white matter and cortical gray matter, and the outer surface was defined as the exterior of the cortical gray matter. As the outer surface was constructed by deforming the inner surface, the two surface meshes are isomorphic, with the same number of vertices and edge connectivity. Due to inter-subject variability of brain shapes, we resampled the surfaces with 40,962 vertices for each hemisphere using our in-house software (Cho et al., 2012). The image preprocessing and cortical thickness computation process were manually checked and corrected by an expert neuroanatomist.

### Noise removal of cortical thickness data

For smoothing cortical thickness data, we adopted the noise removal procedure proposed by Cho et al. (2012) to our problem setting. Cho and his colleagues employed the manifold harmonic transform (MHT) to delineate the cortical thickness data with its spatial frequency components (Vallet and Lévy, 2008). For the transform, the Laplace-Beltrami operator is used to obtain basis functions which results in robustness to noise by filtering out high frequency components (Cho et al., 2012). Since high frequency components of the transformed cortical thickness data were regarded as noise, those components are filtered out, and the cortical thickness data were then reconstructed using only low frequency components (Chung et al., 2007). The cut-off value for the filtering was determined based on goodness of fit. The high frequency components are filtered out: *f*_*x*_, *x* > *D*, where *D* is the cut-off dimension. The cut-off dimension *D* is set by goodness of fit *G*: $G=\overset{N}{\underset{i\;=\;1}{\sum }}{\left||c^{i}-\overset{D}{\underset{k\;=\;1}{\sum }}f_{k}^{i}h^{k}|\right|}^{2}/\overset{N}{\underset{i\;=\;1}{\sum }}{\left||c^{i}|\right|}^{2}$, where *c*^*i*^ is the cortical thickness data of subject *i*, $f_{k}^{i}$ is the *k*th frequency component of subject *i*, and *N* is the number of subjects. We set the cut-off dimension *D* = 1600 resulting in *G* = 0.027 which is conservative value according to the value *G* = 0.05 used in previous study (Qiu et al., 2008; Cho et al., 2012).

### Network construction

To define the nodes in our brain network, we parcellated the cerebral cortex into 68 cortical ROIs based on the Desikan-Killiany Atlas (Desikan et al., 2006). We then computed a connectivity matrix for each subject based on covariance of the cortical thickness data for each pair of ROIs (Step B in Figure 1). The covariance between two ROIs was calculated using Z-score: we considered vertex-wise sampled cortical thicknesses as the distribution for each ROI. We denoted μ(j) and σ(j) as the mean and standard deviation of the cortical thickness data in the j-th ROI, respectively. This enables calculation of the z-score of a cortical thickness value as Z (i, j) = (μ(i)− μ(j))/σ(j). Intuitively, Z (i, j) signifies how much the cortical thickness of the i-th ROI deviates from that of the j-th ROI on average. Similarly, Z(j, i) can be computed using the mean and standard deviation of the i-th ROI. Finally, we defined a symmetric connectivity matrix C(i, j) between nodes iand j as the mean of Z(i, j) and Z(j, i): C(i, j) = ((|Z (i, j)|+ |Z (j, i)|))/2. Here, we used magnitude values of the average z-scores, since the structural connectivity between two ROIs in our network definition measures the extent of similarity in cortical thickness distribution between ROIs.

Since binary networks are simpler to demonstrate and easier to define null model for statistical comparison, we binarized the connectivity matrix C(i, j) using a threshold value for the individual network analysis. Considering appropriate sparsity of the resulting network from previous researches which is approximately ranged from 5 to 25%, we examined a thresholding value in the range of 0.1–0.3 for verification (Achard and Bullmore, 2007; He et al., 2007; Bassett et al., 2008). Edges whose weight below the threshold are binarized to 1 and weight upper the threshold edges are binarized to 0. We then chose 0.2 as the thresholding value and binarized the network which results in 18%. Thresholding value determination and its verification results from the ADNI dataset are shown in the Supplementary section (Supplementary figure 1, Supplementary Threshold selection).

### Individual-level network architecture analysis

We first investigated architectural characteristics of our individual SCNs based on graph-theoretic measures. For each individual subject, the network measures include the degree (the number of edges connected to a node), the clustering coefficient (the number of existing edges divided by number of possible edges from a node's neighbors), the characteristic path length (the average shortest path length from a node to other nodes), and the measure of network small-worldness (C_real_/C_random_)/(L_real_/L_random_) where C is the clustering coefficient and L is the characteristic path length (Watts and Strogatz, 1998; Latora and Marchiori, 2001; Humphries and Gurney, 2008; Rubinov and Sporns, 2010). The random networks were generated by preserving the nodal degree and strength with 100 repetitions for the normalization. Since these scores are not normally distributed, we used the Wilcoxon rank sum test for group comparison of each network measure (Wilcoxon and Wilcox, 1964).

### Group-level network consistency analysis

A group-level network is further constructed to analyze consistency of the individualized SCNs across subjects. For each connection between two ROIs, we counted the number of subjects of which SCN has a binarized edge. We then constructed a binarized group-level network shared in common for more than 50% of the total subjects in the group. Hub nodes from each group-level network were also identified as a node with nodal degree higher than the sum of the mean and standard deviation of total node's degree.

### Network entropy analysis

In order to investigate randomness of the anatomical covariance connectivity, we calculate a nodal entropy for each ROI using the group-level network. In our problem setting, an edge weight in the group-level network was determined based on the number of subjects which has a value 1 for the corresponding edge in the individual SCN. Thus, the normalized edge weight using the total number of nodal degrees for each node can be regarded as the probability of connectedness from one node to other nodes. We therefore calculate the entropy of a node as $\text{H}\left(\text{i}\right)=-\sum_{i}p_{i}\log(p_{i})$. Here, H(i) signifies the nodal entropy which denotes the extent of evenly distributed or the peakiness similar to the work by Raj et al. (2010).

For qualitative comparison between NC and AD groups, we used the normalized nodal entropy values. Since the sparsity of the group-level networks are different between two groups, we calculate the normalized entropy values based on a normal distribution generated by 10,000 random networks for each group. The random network was generated by preserving degrees of every node in the group-level network. The normalized entropy values that are close to 0 indicate that the covariance connectivity of the node to other nodes is closer to the random networks. To investigate the coordinated variance is distributed more randomly AD when compared to NC subjects qualitatively, the 68 ROIs were separated into four sub-groups: Hub A (sustained hub regions in NC and AD group network), Hub B (hub regions in NC group network only), Hub C (hub regions in AD group network only), and non-hub regions. We compared the sub-groups for each NC and AD groups visually.

## Results

### Network architecture analysis

We first validated our network construction method by investigating the network architecture in the resulting SCNs. Clustering coefficients and characteristic path lengths were calculated for the binarized structural connectivity network in an individual level. By considering the network measures of a random network, we obtained a normalized clustering coefficient and normalized characteristic path length (γ = 4.19, λ = 1.63) for each normal control and patient with AD. The result was similar to that observed in previous studies (He et al., 2007; Tijms et al., 2012, 2013), with the resulting small-worldness value (σ = γ/λ = 2.61) implying that the proposed SCNs are small-world.

**Figure 2:**
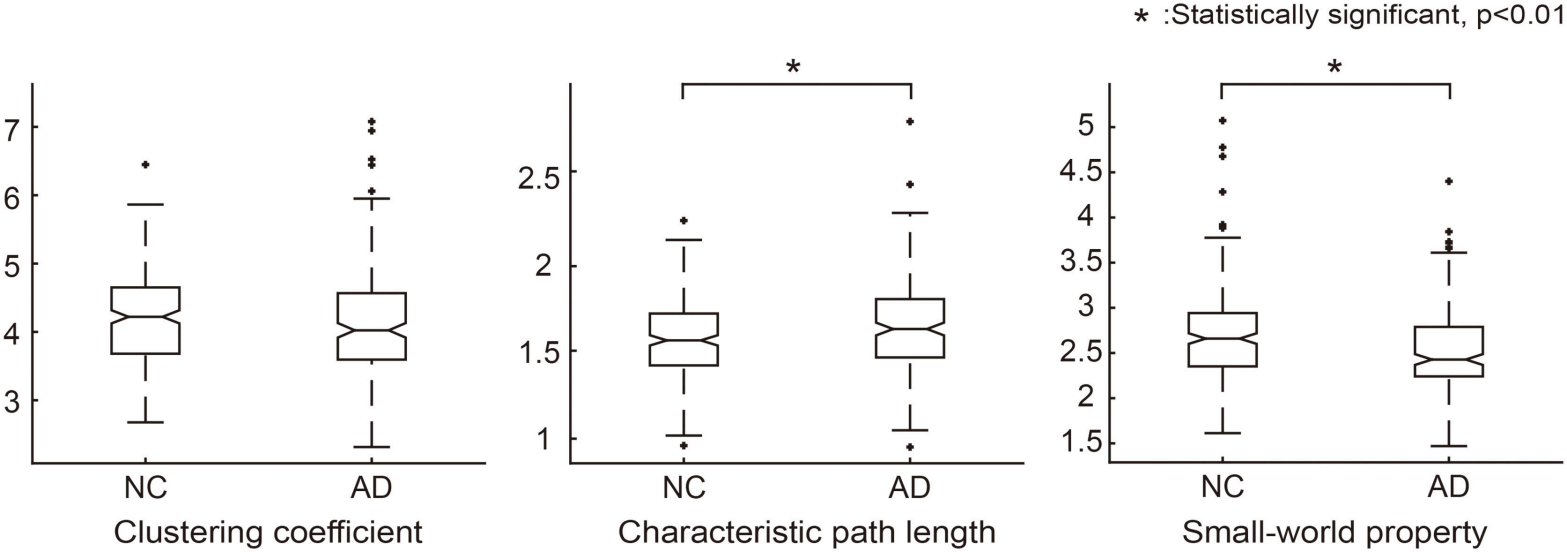
**Graph theoretical measures for group comparison between NC and AD groups**. Normalized clustering coefficients (γ = C_real/C_random), normalized characteristic path lengths (λ = L_real/L_random) and normalized small-world property (σ = γ/λ) were calculated for each individual network. In each graph, the asterisk symbol indicates that the graph measures are significantly different between the groups according to the Wilcoxon rank sum test.

We further analyzed the network architecture for the NC and AD groups separately. Each group had small-world properties and reasonable network sparsity, as shown in Table 2. For comparison purposes, we applied the Wilcoxon rank sum test to each network measure. Characteristic path lengths (*z* = −2.97, *p* < 0.01) and small-worldness values *z* = 4.05, *p* < 0.01) were significantly different between the two groups. Clustering coefficients in AD was smaller than that of NC but there was no significant difference (*z* = 1.81, not significant). Additionally, network sparsity had significant difference between the two groups (*z* = −4.00, *p* < 0.01). Box plots for each group theoretical measures are depicted in Figure 2. The network architecture measures in the ADNI dataset showed the same trend with those of the SMC dataset (Supplementary Table 2, Supplementary Figure 2).

**Table 2:** **Graph theoretical measures of individual network architecture in normal control (NC) and Alzheimer's disease (AD) groups**.

**Dataset**	**Group**	**C_real**	**L_real**	**C_random**	**L_random**	**γ**	**λ**	**σ**	**s**
SMC	NC	0.95	3.23	0.23	2.02	4.23	1.60	2.69	0.17
	AD	0.94	3.32	0.23	1.99	4.14	1.67	2.51	0.18

*Normalized clustering coefficients (γ = C_real/C_random), normalized characteristic path lengths (λ = L_real/L_random) and normalized small-world property (σ = γ/λ) were calculated for each individual structural covariance network (SCN). Mean values of NC and AD group for the graph theoretical measures are obtained*.

*SMC: Samsung Medical Center, C: clustering coefficient, L: characteristic path length, real: result obtained from binarized structural connectivity network, random: result obtained from random network, γ: normalized clustering coefficient, λ: normalized characteristic path length, σ: small-world property, s: sparsity of network*.

### Group-level network consistency analysis

**Figure 3:**
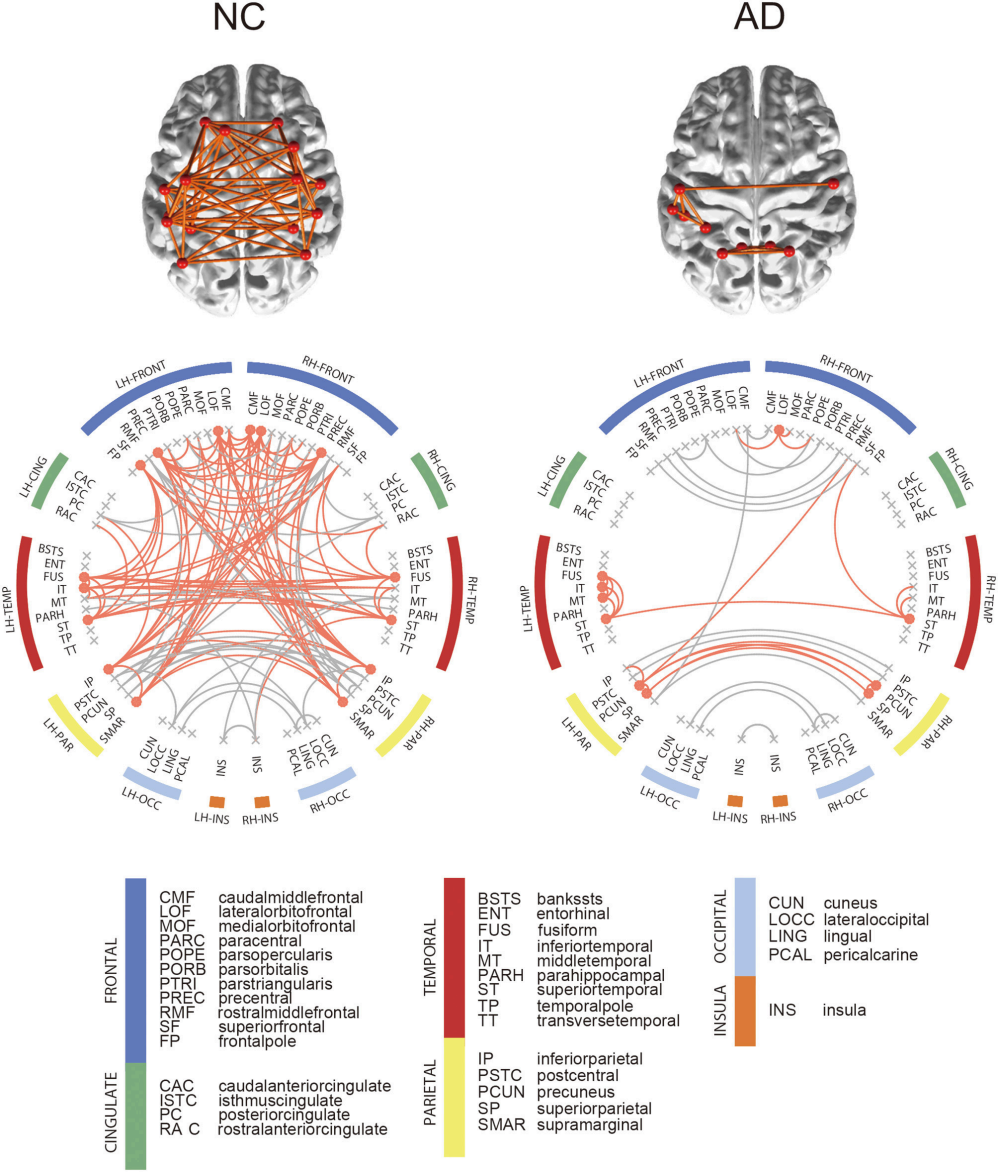
**Connectograms of binarized group-level network**. In the connectogram, hub regions and their connections were illustrated in orange color. Among all network edge, only hub connections were depicted on brain. Hub regions were obtained from each binarized group-level network, which are nodes with nodal degree higher than the sum of the mean and standard deviation of total node's degree.

For each group, we computed the group-level network and identified hub regions in the networks. The NC group had 190 edges shared by more than 50% of NC subjects, and 15 brain regions were identified as hubs in the network. Figure 3 shows the connectogram for the group-level network and its corresponding display of the brain connectivity superimposed on the template brain surface. In this figure, the connectivity was displayed only for the identified hub regions. The 5 top-ranked hub regions in the NC group include left inferior parietal cortex, left precentral gyrus, left supramarginal gyrus, right inferior parietal cortex, right precentral gyrus. We also found 104 edges for the AD group and identified 10 brain regions as hubs. Among the hub regions in the AD group, 5 hubs were also identified as hubs in the NC group: left fusiform gyrus, left inferior temporal gyri, left superior temporal gyrus, right lateral orbitofrontal cortex, and right superior temporal gyrus. Although the AD patients have significantly higher sparsity in the individual SCNs compared to the NC subjects, the consistent edges in the NC group across subjects is twice more dense than that of the AD group as shown in the group-level networks. All the hub regions are listed in Supplementary Table 1. The results were consistent for both SMC and ADNI datasets.

### Network entropy analysis

**Figure 4:**
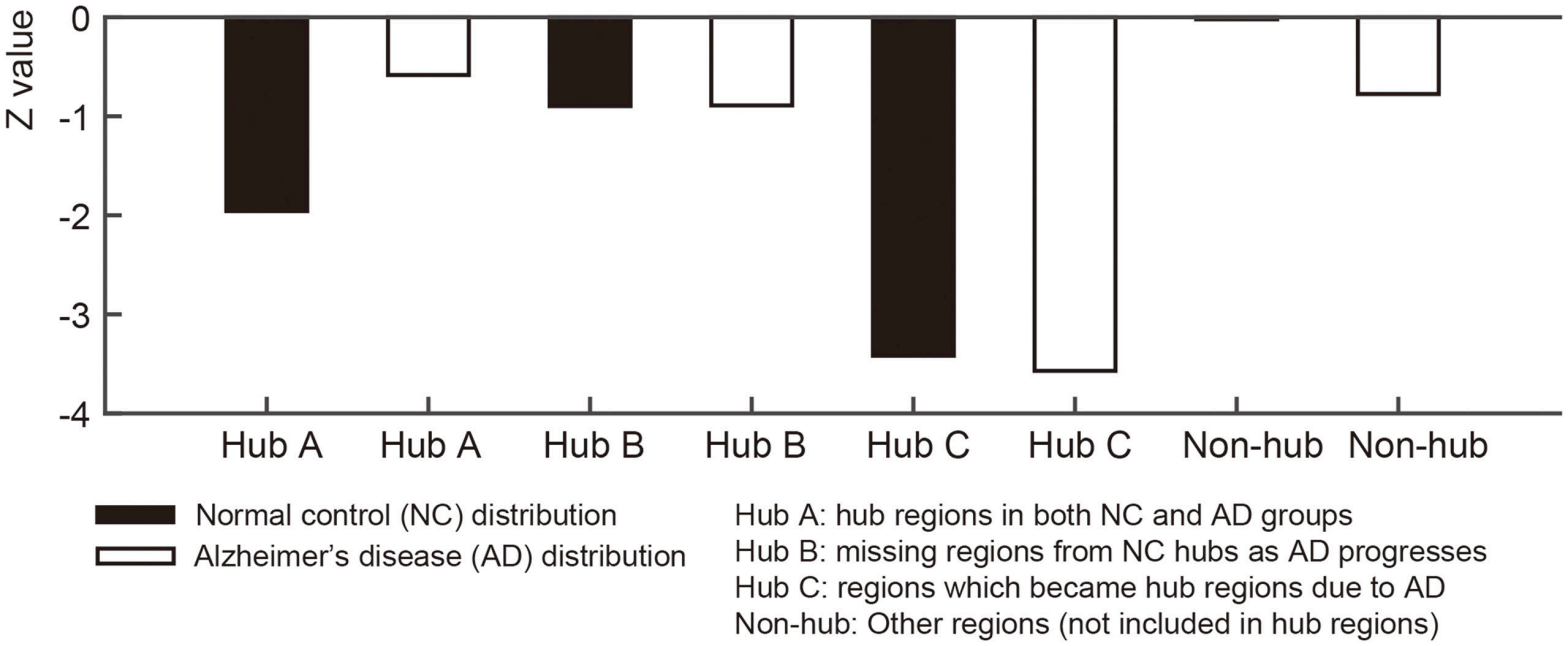
**Figures illustrate bar graphs of average nodal entropy ***z***-value of four regions groups (Sustained, disrupted, emerged, and other regions)**. Nodal entropy *z*-value of group-level network is calculated using 10,000 degree preserved random network. Hub A: hub regions from normal control (NC) group-level network hubs which existed in hub regions of Alzheimer's disease (AD) group-level network. Hub B: missing regions from NC group-level network's hub regions as AD progresses. Hub C: regions from AD group-level network's hub regions which became hub regions according to the disease. Non-hub: Other regions which were not included in hub regions.

For each node in the group-level network, we calculated the nodal entropy in order to investigate randomness of the anatomical covariance connectivity in the NC and AD groups. Nodal entropy of the AD group-level network was higher than that of the NC group in all brain regions. We further classified the 68 ROIs into four sub-groups: Hub A (sustained hub regions), Hub B (NC only hub regions), Hub C (AD only hub regions), and non-hub regions. Figure 4 shows the bar graph of the mean *z*- values for the four sub-groups of each dataset. As explained in the method section, the *z*-value of entropy was calculated based on the random distribution of entropy values in the random networks, and thus smaller absolute *z*-values represent increased randomness in the covariance connectivity of the corresponding node. The normalized entropy for each group was compared qualitatively. As shown in Figure 4, the absolute *z*-value of the Hub A is much smaller in the AD group compared to that of the NC group. Also, in the NC group, the entropy of the Hub B is higher than that of the Hub A, while the result is opposite in the AD group. The entropy of the Hub C is much smaller for both NC and AD groups when compared to the other hub regions. Lastly, we observed that every hub region have smaller entropy than the other non-hub regions for both groups. The results were similar for the ADNI dataset as shown in Supplementary results (Supplementary Network architecture analysis to Network entropy analysis).

## Discussion

In this study, we presented a new method to construct individual SCNs based on cortical thickness covariance, and applied the proposed approaches to a large group of AD patients. We first demonstrated that the AD patients had significantly disrupted network architecture when compared to NC subjects, which implies that the anatomical covariance connectivity exhibited more spreading out and therefore inefficient integration in AD patients. We further showed the coordinated variance of cortical thickness in different brain regions is distributed more randomly in AD patients by investigating nodal entropy in the SCNs. As such, we hypothesize that the structural covariance connectivity becomes disorganized across the brain regions in AD, while the NC subjects maintain more clustered and consistent coordination in cortical atrophy variations.

In our study, structural connectivity was defined based on the similarity of surface-based cortical thickness data between two brain regions, i.e., if two regions had a certain extent of similarity in terms of cortical thickness, then we connected them, thereby generating a structural covariance network. The connectivity in our network could therefore be used to quantitatively measure the amount of correlated change in different regions of the cerebral cortex. Note that the connectivity in our network should be interpreted from the statistical perspective rather than a real anatomical connection. Similarly as in the previous work on SCNs (Dai et al., 2011, 2013; Evans, 2013; Raamana et al., 2014, 2015), our covariance networks indeed represent coordinated variance of cortical thickness between two brain regions by encoding both the difference of mean cortical thickness and its variance in a ROI. While there have been a number of cortical thickness-based studies conducted, most have been limited to regional studies investigating cortical atrophy in a specific region. These regional analyses have been successfully applied to not only normal aging but also several neurodegenerative brain diseases including AD, and have shown that cortical atrophy in specific brain areas is associated with both normal aging and degenerative diseases (Salat et al., 2004; Sowell et al., 2004; Lerch et al., 2008; Tae et al., 2008; Bernhardt et al., 2009b; Querbes et al., 2009; Koolschijn et al., 2010; Järnum et al., 2011). It has been asserted that the occurrence of cortical atrophy in some brain regions may influence the rate of atrophy in other regions. However, it remains to be elucidated how such simultaneous cortical atrophy occurs in normal aging or neurodegenerative brain diseases. Using our definition of network connectivity, the proposed SCN can provide surface-based measures to investigate the interaction between two brain regions in terms of cortical atrophy. It is currently unclear whether related changes in cortical regions occur in gray matter thickness as a result of AD pathology, however, our method for constructing individual SCNs provides a quantitative measure for such studies of coordinated variations in cortical atrophy in AD patients.

Our method has an inherent advantage in that it can be used to investigate the individual characteristics of network architecture. Every individual in the NC and AD groups exhibited a small-world property in the resulting SCNs. Moreover, we further observed that the AD patients had significantly disrupted network architecture when compared to NC subjects (the patients with AD had smaller clustering coefficients and larger characteristic path lengths than their NC counterparts, leading to disrupted small-worldness in the AD group). We speculate that the disrupted network architectural properties are caused by “spreading out” of the structural connectivity, as opposed to clustering. This could imply that the coordinated similarity of cortical thickness between brain regions is disrupted in AD due to selective cortical atrophy in AD patients. The results of the hub analysis showed similar characteristics in the AD patients. For the NC subjects, 15 regions were identified as hub regions. In AD, 10 regions were distinguished. We could investigate that number of hubs regions is decreased and some of them are changed to another region despite higher SCN sparsity in AD group. This deterioration of the hub regions in AD is clearly associated with disrupted structural covariance in terms of both local segregation (clustering coefficients) and global integration (characteristic path length) as shown in our network architecture analysis. We applied the methods to the ADNI dataset and obtained similar results.

We calculated nodal entropy of the group-level networks to investigate randomness of the anatomical covariance connectivity. Based on the work by Raj and his colleagues, a network entropy captures the peakiness or the extent of uniformly distributed connectivity. Higher value of nodal entropy denotes that the structural connections to other regions become more random. Such a node with high nodal entropy will have randomly connected network edges, of which connections are not specific across brain regions. In our experiment, most regions had higher entropy in AD except the left and right caudal anterior cingulate gyri. The increased entropy in AD would be able to support the results that the SCN connectivity becomes inefficient despite higher sparsity. By extension, the normalized nodal entropy values were also used for statistical analysis between groups. As the absolute value is closer to zero, it indicates more random since the network is more similar to random networks. The brain regions were separated into four groups to investigate the different characteristics of the hub regions from other regions for each group. The bar graph in Figure 4 shows Hub A, the sustained hub in both groups, is more random in NC than AD group. In other words, Hub A connections to other regions are scattered however the regions play a key role as hub in spite of AD progression. Hub B is more random-like than Hub A in NC and Hub A has more randomness in AD. The results imply that connections of the NC hub regions become more spread and scattered rather than clustered or efficient in AD. Compare to the hub regions, the non-hub regions are close to zero which means they are approximately random. These results are in line with the results of the ADNI dataset.

We acknowledge some limitations in our study. First, we did not thoroughly address individual variability in SCNs. Some individuals had deviated values in the graph theoretical measures as shown in Figure 2 which depicts the graph theoretic measures for each group. Although the overall network properties follow a certain trend that shows small-worldness, such as large clustering coefficients and small characteristic path lengths, detailed analysis of the individual characteristics remains challenging. Second, although our individual SCNs have small-worldness properties in terms of network architecture, the network seems somewhat different from the diffusion tensor imaging (DTI)-based structural networks. The proposed SCNs are based on correlations of structural properties in gray matter, while the DTI-based approach relies on the strength of white matter connectivity between two gray matter regions. Cortical thinning in two different gray matter regions may be associated with the change of white matter connections between them. Therefore, a promising direction for future work will be to investigate the relationship between the proposed SCNs and other types of network, based on a variety of image modalities. Third, it would be misleading to understand structural covariance. The term ‘structural covariance network (SCN)’ is inspired by the review paper (Evans, 2013). Inspired by the review paper, recently some SCNs have been proposed based on the difference of mean cortical thickness between ROIs (Dai et al., 2011, 2013; Raamana et al., 2014, 2015). Our SCNs are based on both the difference of mean cortical thickness and its variance. Thus, we believe that the proposed method for edge weight computation reveals the amount of covariance of the cortical thickness between ROIs. Fourth, we could use the individual SCNs fully. In this paper, we sought to understand AD using structural covariance. We have actually analyzed network architectural properties in both NC and AD groups using the individualized networks. The graph theoretical measures were computed individually using individualized networks and enabled statistical analysis. We would use the individual SCNs in prediction correlation study with cognitive scores for future works. The individual SCNs would be useful feature for predicting group membership or enable practical interpretation by combining with cognitive data.

Another possible limitation of this study is the binarization. We binarized networks since the networks are simpler to demonstrate and easier for statistical comparison. However, it has some limitations in that the binarization could cause information loss. Weighted networks provide more information about the relationship between nodes. The networks would be useful in studying local study since each node gives weight information. In addition, many researchers is still debating about the choice of a threshold. Since it has no golden rule, binarized networks could be unclear to understand compare to weighted networks.

## Author contributions

HJinK, DN, and SS assembled the data. HJongK, JKS, and SS designed the experiments. HJongK performed the experiments and prepared the manuscript. JKS and SS supervised the experiments and edited the manuscript. JHS and CH gave support and advice. Data used in preparation of this article were obtained from the Alzheimer's Disease Neuroimaging Initiative (ADNI) database (adni.loni.usc.edu). As such, the investigators within the ADNI contributed to the design and implementation of ADNI and/or provided data but did not participate in analysis or writing of this report. A complete listing of ADNI investigators can be found at: http://adni.loni.usc.edu/wp-content/uploads/how_to_apply/ADNI_Acknowledgement_List.pdf.

### Conflict of interest statement

The authors declare that the research was conducted in the absence of any commercial or financial relationships that could be construed as a potential conflict of interest.
