# Supplementary material for: Using Individualized Brain Network for Analyzing Structural Covariance of the Cerebral Cortex in Alzheimer's Patients
Source: Front Neurosci. 2016 Sep 1;10:394. doi: 10.3389/fnins.2016.00394 (PMC5007703; doi:10.3389/fnins.2016.00394)
Supplement: Supplementary file 1 [file Presentation1.PDF]

frontiers

Supplementary Material

Using individualized brain network for analyzing structural covariance of the cerebral cortex in Alzheimer's patients

Hee-Jong Kim, BE,[1,2] Jeong-Hyeon Shin, BE,[1,2] Cheol E. Han, PhD,[1,2] Hee Jin Kim, MD, Ph.D,[3,4] Duk L. Na, MD, Ph.D,[3,4] Sang Won Seo, MD, Ph.D,[3,4,*] Joon-Kyung Seong, PhD,[1,2,*] for the Alzheimer's Disease Neuroimaging Initiative[a]

[1]School of Biomedical Engineering, Korea University, Seoul, Republic of Korea

[2]Department of Bio-convergence Engineering, Korea University, Seoul, Republic of Korea

[3]Department of Neurology, Sungkyunkwan University School of Medicine, Samsung Medical Center, Seoul, Republic of Korea

[4]Neuroscience Center, Samsung Medical Center, Seoul, Korea

[a]Data used in preparation of this article were obtained from the Alzheimer's Disease Neuroimaging Initiative (ADNI) database (adni.loni.usc.edu). As such, the investigators within the ADNI contributed to the design and implementation of ADNI and/or provided data but did not participate in analysis or writing of this report. A complete listing of ADNI investigators can be found at: http://adni.loni.usc.edu/wp-content/uploads/how_to_apply/ADNI_Acknowledgement_List.pdf

***Correspondence:**
Joon-Kyung Seong, PhD
Address: School of Biomedical Engineering, Korea University, 145, Anam-ro, Seongbuk-gu, Seoul, Republic of Korea
Phone: +82-2-3290-5660
E-mail: jkseong@korea.ac.kr

Sang Wong Seo, MD, PhD
Address: Department of Neurology, Sungkyunkwan University of Medicine, Seoul, 50 Irwon-dong, Gangnam-gu, Seoul 135-710, Republic of Korea.
Phone: +82-2-3410-1233
Fax: +82-2-3410-0052
E-mail: sangwonseo@empal.com

1 Supplementary figures

Supplementary figure 1. Graph theoretical measures sorted by threshold values for both normal control (NC) and Alzheimer's disease (AD) groups separately. Normalized clustering coefficients (γ=C_real/C_random), normalized characteristic path lengths (λ=L_real/L_random) and normalized small-world property (σ= γ/ λ) were calculated for each individual network. Over a thresholding value range from 0.1 to 0.3, graph theoretical measures were obtained. In each graph, the asterisk symbol indicates that the graph measures are significantly different between the groups according to the Wilcoxon rank sum test.

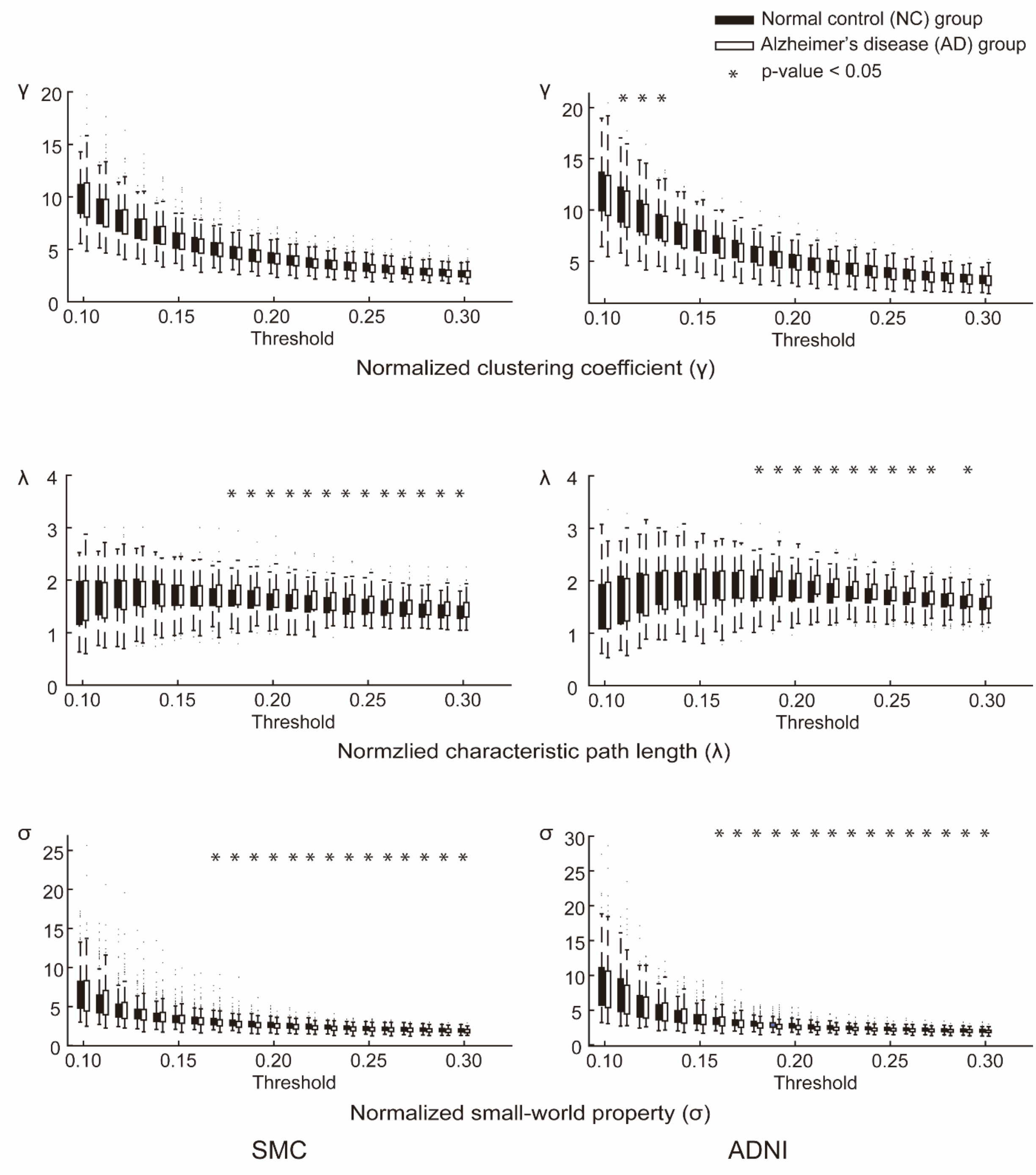

Note: SMC: Samsung Medical Center dataset, ADNI: Alzheimer's Disease Neuroimaging Initiative dataset, C: clustering coefficient, L: characteristic path length, real: result obtained from binarized structural connectivity network, random: result obtained from random network, γ: normalized clustering coefficient, λ: normalized characteristic path length, σ: small-world property, s: sparsity of network.

Supplementary figure 2. Graph theoretical measures for group comparison between normal controls (NC) and Alzheimer's disease (AD) groups using the ADNI dataset. Normalized clustering coefficients (γ=C_real/C_random), normalized characteristic path lengths (λ=L_real/L_random) and normalized small-world property (σ= γ/ λ) were calculated for each individual network. In each graph, the asterisk symbol indicates that the graph measures are significantly different between the groups according to the Wilcoxon rank sum test.

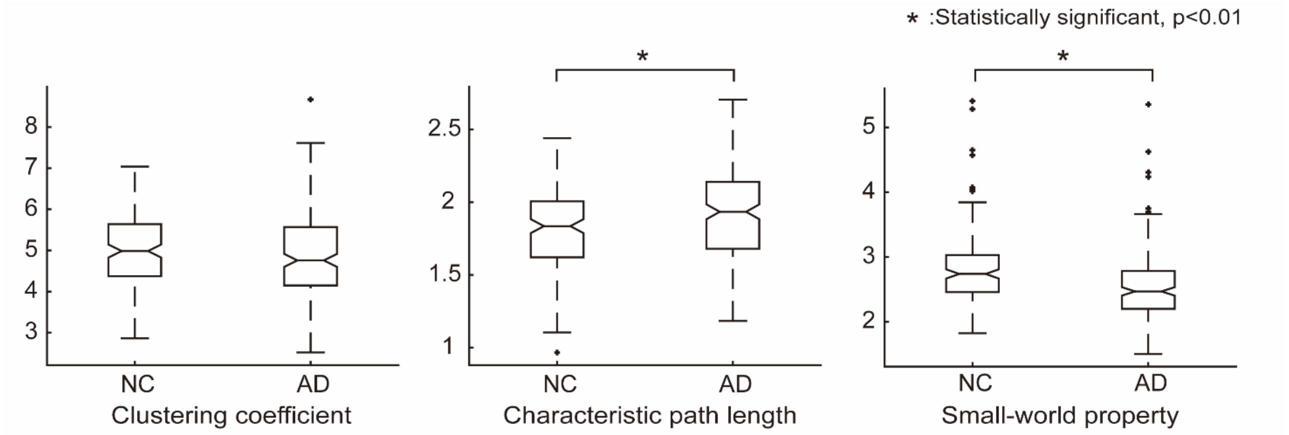

Supplementary figure 3. Connectograms of binarized group-level network using the ADNI dataset. In the connectogram, hub regions and their connections were illustrated in orange color. Among all network edge, only hub connections were depicted on brain. Hub regions were obtained from each binarized group-level network, which are nodes with nodal degree higher than the sum of the mean and standard deviation of total node's degree.

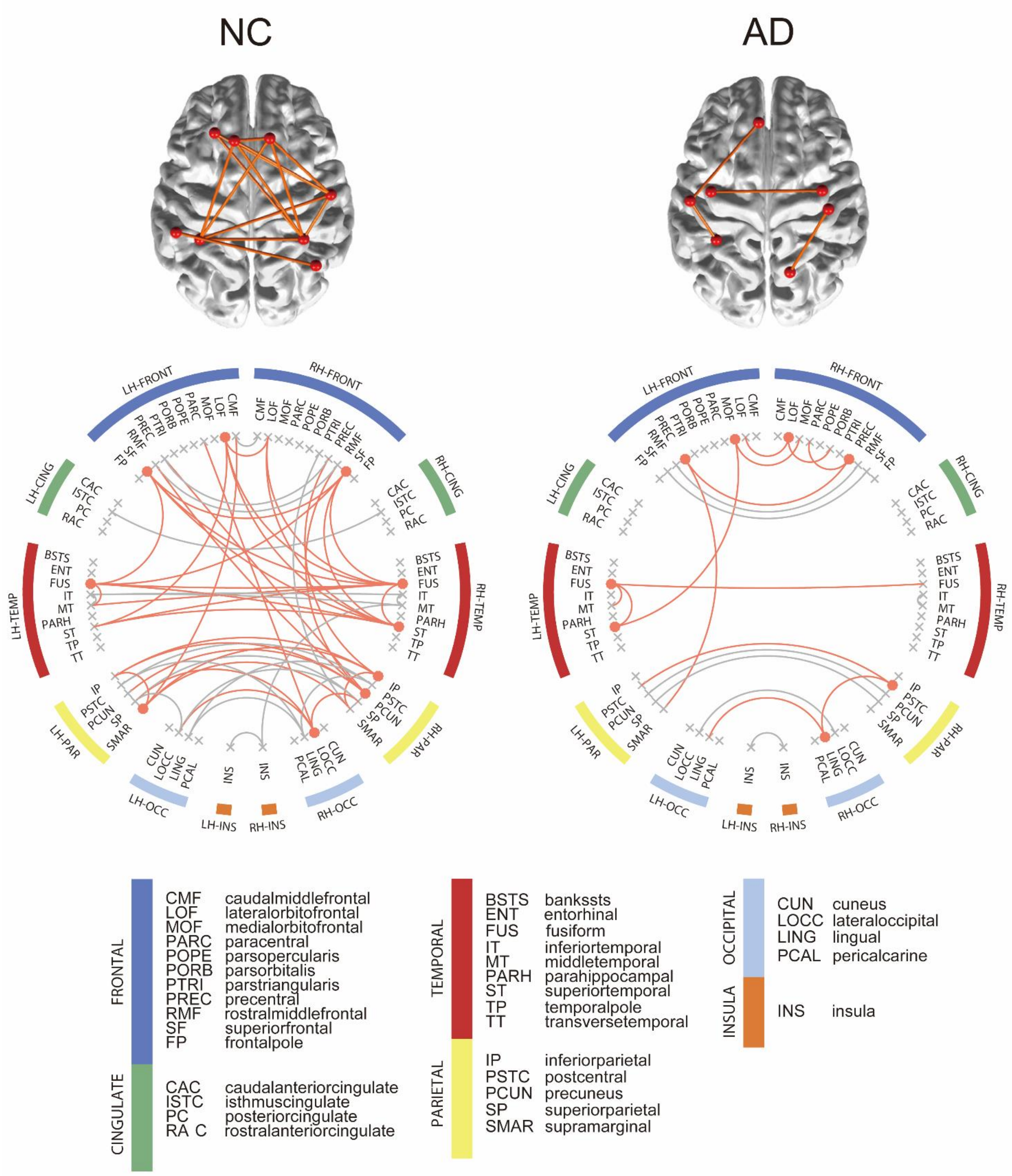

Supplementary figure 4. Figures illustrate bar graphs of average nodal entropy z value of four regions groups using the ADNI dataset (Sustained, disrupted, emerged and other regions). Nodal entropy z value of group-level network is calculated using 10000 degree preserved random network. Hub A: hub regions from normal control (NC) group-level network hubs which existed in hub regions of Alzheimer's disease (AD) group-level network. Hub B: missing regions from NC group-level network's hub regions as AD progresses. Hub C: regions from AD group-level network's hub regions which became hub regions according to the disease. Non-hub: Other regions which were not included in hub regions.

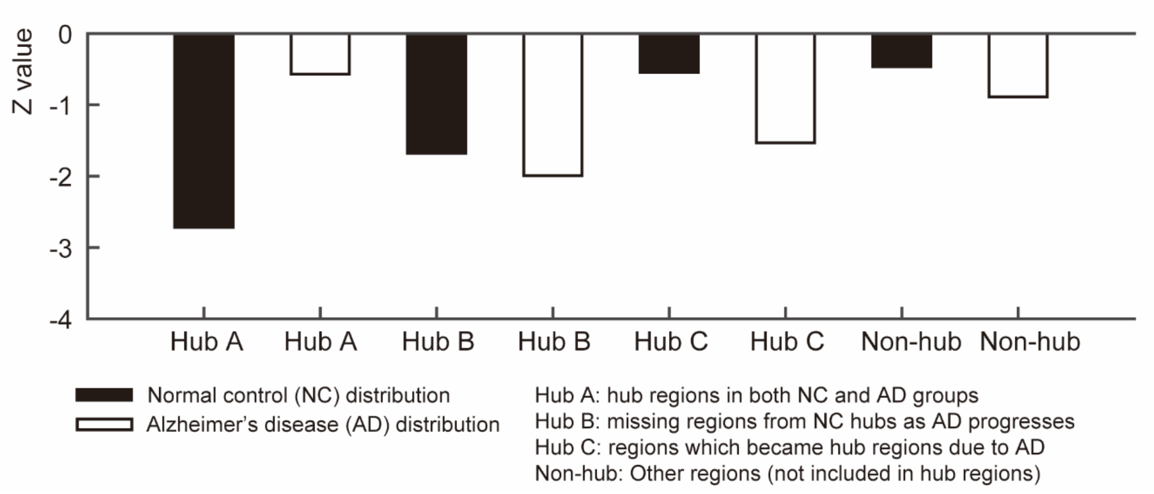

2 Supplementary tables

Supplementary table 1. Hub regions from group-level network. Hub regions are obtained from group-level network. The group-level network are the network having edges shared by more than 50 % of subjects in the group.

SMC	
NC	AD
left fusiform gyrus	left fusiform gyrus
left inferior parietal cortex	left inferior temporal gyri
left inferior temporal gyri	left middle temporal gyrus
left lateral orbitofrontal cortex	left precuneus cortex
left precentral gyrus	left superior parietal cortex
left superior frontal gyrus	left superior temporal gyrus
left superior temporal gyrus	right lateral orbitofrontal cortex
left supramarginal gyrus	right precuneus cortex
right caudal middle frontal gyrus	right superior parietal cortex

right fusiform gyrus	right superior temporal gyrus
right inferior parietal cortex	
right lateral orbitofrontal cortex	
right precentral gyrus	
right superior temporal gyrus	
right supramarginal gyrus	
ADNI	
NC	AD
left fusiform gyrus	left fusiform gyrus
left lateral orbitofrontal cortex	left medial orbital frontal cortex
left superior frontal gyrus	left precentral gyrus
left supramarginal gyrus	left superior temporal gyrus
right fusiform gyrus	right lateral orbitofrontal cortex
right inferior parietal cortex	right lingual gyrus
right lateral occipital cortex	right postcentral gyrus
right precuneus cortex	right precentral gyrus
right superior frontal gyrus	
right superior temporal gyrus	

Supplementary table 2. Graph theoretical measures of individual network architecture in normal control (NC) and Alzheimer's disease (AD) groups using ADNI dataset. Normalized clustering coefficients (γ=C_real/C_random), normalized characteristic path lengths (λ=L_real/L_random) and normalized small-world property (σ= γ/ λ) were calculated for each individual structural covariance network (SCN). Mean values of NC and AD group for the graph theoretical measures are obtained.

Dataset	Group	C_real	L_real	C_random	L_random	γ	λ	σ	s
**ADNI**	NC	0.95	3.75	0.20	2.06	5.00	1.82	2.79	0.16
	AD	0.94	3.90	0.20	2.03	4.85	1.92	2.56	0.17

Note: SMC: Samsung Medical Center, ADNI: Alzheimer's Disease Neuroimaging Initiative, C: clustering coefficient, L: characteristic path length, real: result obtained from binarized structural connectivity network, random: result obtained from random network, γ: normalized clustering coefficient, λ: normalized characteristic path length, σ: small-world property, s: sparsity of network.

3 Supplementary results

3.1 Threshold selection

For all subjects in the SMC and ADNI datasets, the individual SCNs were constructed using the respective cortical thickness data. When binarizing a network, the selection of a threshold affects the properties of the network (Drakesmith et al. 2015). We considered appropriate sparsity of the resulting network from previous researches, which is ranged from 5-25%. In our individual SCNs, we evaluated the network properties for a threshold range from 0.1 to 0.3 at intervals of 0.01 considering both sparsity and the small-worldness value of the network (Achard and Bullmore 2007; Bassett et al. 2008; He, Chen, and Evans 2007). For the NC group, the sparsity ranges from 10%/9% to 25%/23% while it was from 10%/9% to 26%/24% for the AD group (SMC/ADNI). The network sparsity itself was significantly different between two groups (p-value<0.01(SMC)/p-value<0.01(ADNI)). Edges whose weight below the threshold are binarized to 1 and weight upper the threshold edges are binarized to 0.

We further examined network architectural characteristics and between-group differences over a thresholding value range from 0.1 to 0.3 for verification. The middle of the range (0.2) was applied for binarizing to all networks whose sparsity is similar to previous cortical thickness network studies which results in 18% sparsity in SMC dataset and 16% sparsity in ADNI dataset (He et al. 2007; Sporns and Zwi n.d.). However, all the networks around 0.2 had similar patterns for clustering coefficients, characteristic path lengths, and small-worldness (Supplementary figure 1). There were also similar tendencies for the group comparisons between NC and AD groups results around 0.2 in particular. Both the SMC dataset and the ADNI dataset had the similar results for varying threshold values.

3.2 Network architecture analysis

From this section, we report results in the ADNI dataset using our method described in main text. The results showed the same trends with the SMC dataset overall.

We applied the same network architecture analysis to the ADNI dataset. The network architecture measures showed similar results to results from the SMC dataset. Box plots for each group theoretical measures are shown in Supplementary Figure 2. We first validated the network architecture in the resulting SCNs, which are clustering coefficients and characteristic path lengths. We obtained a normalized clustering coefficient and normalized characteristic path length (γ=4.92, λ=1.87) with the resulting small-worldness value ($\sigma=\gamma/\lambda$= 2.66). Same as the method in main text, we further investigated for the NC and AD groups. As shown in Supplementary table 2, NC and AD groups had small-world properties and network sparsity which are in line with results from the SMC dataset. For group comparison, Characteristic path lengths (z=-2.94, p<0.01) and small-worldness values (z=4.68, p<0.00001) were significantly different between the two groups. Same as the SMC results, clustering coefficients had no significant difference (z=1.79, not significant) but was smaller in AD than that of NC group. Group difference for network sparsity (z=-3.05, p<0.01) was also consistent with the SMC results.

3.3 Group-level network consistency analysis

For the ADNI dataset, the group-level networks showed the similar trend to that of the SMC dataset (Supplementary figure 3). The group-level network of the NC group consists of 55 edges with 10 hub regions, while the AD group-level network has 20 edges and 8 hub regions. Despite higher

sparsity in the individual SCNs in AD compare to NC group, the consistent edges were denser in the NC group. The results were consistent with those of SMC dataset. All the hub regions are listed in Supplementary Table 1.

3.4 Network entropy analysis

The ADNI dataset showed that 66 brain regions out of 68 ones have higher entropy value in the AD group: the left and right caudal anterior cingulate gyri recorded lower entropy values in the AD group. The results were similar for the ADNI dataset as shown in Supplementary figure 4. Same as the entropy analysis in the main text, we classified the ROIs into four sub-groups: Hub A (sustained hub regions), Hub B (NC only hub regions), Hub C (AD only hub regions), and non-hub regions. The bar graph of the mean z values for the sub-groups of each dataset is depicted in Supplementary figure 4. The overall results are similar to results from the SMC dataset.

4 References

Achard, Sophie and Ed Bullmore. 2007. "Efficiency and Cost of Economical Brain Functional Networks." *PLoS Comput Biol* 3(2):e17–e17.

Bassett, Danielle S. et al. 2008. "Hierarchical Organization of Human Cortical Networks in Health and Schizophrenia." *The Journal of Neuroscience* 28(37):9239–48.

Drakesmith, M. et al. 2015. "Overcoming the Effects of False Positives and Threshold Bias in Graph Theoretical Analyses of Neuroimaging Data." *Neuroimage*.

He, Yong, Zhang J. Chen, and Alan C. Evans. 2007. "Small-World Anatomical Networks in the Human Brain Revealed by Cortical Thickness from MRI." *Cerebral cortex* 17(10):2407–19.

Sporns, Olaf and Jonathan D. Zwi. n.d. "The Small World of the Cerebral Cortex." *Neuroinformatics* 2(2):145–62. Retrieved (http://dx.doi.org/10.1385/NI:2:2:145).
